# Retraction: Long non-coding RNA SNHG1 protects human AC16 cardiomyocytes from doxorubicin toxicity by regulating miR-195/Bcl-2 axis

**DOI:** 10.1042/BSR-2019-1050_RET

**Published:** 2023-01-30

**Keywords:** apoptosis, cardiotoxicity, doxorubicin, long non-coding RNA SNHG1, niR-195

This article is being retracted from *Bioscience Reports* at the request of the Editor-in-Chief and the Editorial Board following receipt of a notification from a reader, alerting the Editorial Board to multiple similarities between the western blots of this manuscript and those of other articles. The GAPDH bands from Figure 3F are similar to those found in Fig. 6A A549/GAPDH from Wang et al. 2019 (doi: 10.1042/BSR20182433) and Fig. 4C AMC-HN-8/GAPDH from Li et al. 2019 (doi: 10.1042/BSR20181882). The authors have been contacted with regards to the retraction and have not responded to the Journal's queries or the concerns raised. Given the extent of the issues raised, the Editorial Board stand by the decision to retract the article.

